# Glycyrrhetinic Acid-Poly(ethylene glycol)-glycyrrhetinic Acid Tri-Block Conjugates Based Self-Assembled Micelles for Hepatic Targeted Delivery of Poorly Water Soluble Drug

**DOI:** 10.1155/2013/913654

**Published:** 2013-11-25

**Authors:** Fengbo Wu, Ting Xu, Chi Liu, Can Chen, Xiangrong Song, Yu Zheng, Gu He

**Affiliations:** ^1^Department of Pharmacy and State Key Laboratory of Biotherapy, West China Hospital, Sichuan University, No. 37 Guoxue Alley, Chengdu, Sichuan 610041, China; ^2^The First Affiliated Hospital of Chengdu Medical College, Chengdu, Sichuan 610500, China

## Abstract

The triblock 18**β**-glycyrrhetinic acid-poly(ethylene glycol)-18**β**-glycyrrhetinic acid conjugates (GA-PEG-GA) based self-assembled micelles were synthesized and characterized by FTIR, NMR, transmission electron microscopy, and particle size analysis. The GA-PEG-GA conjugates having the critical micelle concentration of 6 × 10^−5^ M were used to form nanosized micelles, with mean diameters of 159.21 ± 2.2 nm, and then paclitaxel (PTX) was incorporated into GA-PEG-GA micelles by self-assembly method. The physicochemical properties of the PTX loaded GA-PEG-GA micelles were evaluated including *in vitro* cellular uptake, cytotoxicity, drug release profile, and *in vivo* tissue distribution. The results demonstrate that the GA-PEG-GA micelles had low cytotoxicity and good ability of selectively delivering drug to hepatic cells *in vitro* and *in vivo* by the targeting moiety glycyrrhetinic acid. In conclusion, the GA-PEG-GA conjugates have potential medical applications for targeted delivery of poor soluble drug delivery.

## 1. Introduction

18*β*-glycyrrhetinic acid (GA, 3*β*-hydroxyl-11-oxo-olean-12-ene-29-oic acid, **1**) is one of the main active principles of the plant *Glycyrrhizae radix*, and GA is the hydrolysis active product of glycyrrhizic acid. GA has been used as medicine to treat allergic and hepatic diseases [1, 2]. GA suppresses the tumor promoting effect of 12-O-tetradecanoylphorbol-13-acetate (TPA) and 7,12-dimethylbenz[a]anthracene on skin tumor formation in mice [3] and has antitumor activities [4]. Recently, it has been shown that GA could induce apoptosis in human hepatoma, leukemia, and gastric cancer cells at high concentrations [5]. But, concomitantly, GA exhibited increased scarce stability and poor water solubility resulting in a decreased therapeutic index. 

In the past few years, several research groups reported that the GA modified drug delivery systems bearing good hepatoma cellular targeted efficiency. Mao et al. found that the cellular uptake of liposomes modified with glycyrrhetinic acid by rat hepatocytes was 3.3-fold higher than that of unmodified ones [6]. Recently, Yuan and collaborators prepared chitosan nanoparticles, alginate nanoparticles, PLGA, and poly(ethylene glycol)-b-poly(gamma-benzyl L-glutamate) micelles modified with glycyrrhizic acid and confirmed that these modified nanoparticles or micelles preferentially accumulated in rat hepatocytes by a ligand-receptor interaction [7–17]. Tian et al. also reported a liver-targeted drug delivery carrier, composed of chitosan/poly(ethylene glycol)-glycyrrhetinic acid nanoparticles, prepared by an ionic gelation process, in which glycyrrhetinic acid acted as the targeting ligand [18]. In our previous study, we constructed glycyrrhetinic acid-modified stealth cationic liposomes loaded with pDNA and they were found to transfect human hepatocellular carcinoma cell line HepG2 with high efficiency [19]. Recently, the possibility of synthesizing a number of polyethylene glycol esters of GA and characterizing them to assess their chemical and enzymatic hydrolysis has been investigated [20–23]. But these polymer-drug conjugates have been designed as potential dermal prodrugs, and the short hydrophilic chain of oligoethylene esters cannot provide self-assembly properties. However, so far, there has been no consensus regarding which strategy provides the optimal strategy outcome. In our recent study, biodegradable GA-mPEG conjugates are synthesized by poly (ethylene glycol) monomethyl ether (mPEG). The mGA-mPEG was synthesized through succinic anhydride chain which was used as a bridge for the attachment of GA with polyethylene glycol monomethyl. Meanwhile, GA-mPEG was synthesized without succinic anhydride chain. According to our results, there is no significant difference between the mGA-mPEG and the GA-mPEG micelles in their physicochemical properties, when they were formed under identical conditions [24]. And Tian et al. reported that there is no significant difference between the CTS/GA-PEG-GA NPs and the CTS/PEG-mGA NPs in their ability to target the liver, when they were formed under identical conditions. This indicated that the C3-hydroxyl group in GA has little influence on the targeting ability [25]. Although there had been demonstrated successes in deploying glycyrrhetinic acid as targeted group on hepatoma targeted delivery, however, many GA modified drug delivery systems were complicate, and some components were expensive or instable in room temperature.

In our most recent study, GA could become the hydrophobic fragment of GA-mPEG conjugates, and the conjugates could form self-assembly micelles bearing low cytotoxicity to HEK293 cell line [24]. Tian et al. reported the mixture of single and double modified GA-PEG conjugates that showed good ability to target the liver [25]. 

In the current study, the convenient, economic, and effective methods to prepare hepatoma targeting polymeric micelles, the surfaces of which are anchored with GA, have been successfully developed for drug delivery to the hepatoma cells. The aim of this paper was the synthesis of structurally uniformed glycyrrhetinic acid-poly(ethylene glycol)-glycyrrhetinic acid triblock conjugate (GA-PEG-GA) and to develop an intravenous formulation of paclitaxel utilizing nanomicellar technology to increase water solubility of PTX. GA-PEG-GA with controllable quality was easily synthesized in large amounts on an industrial scale and seemed to be a potent candidate as micellar carrier for PTX entrapment.

## 2. Materials and Methods

### 2.1. Materials

18*β*-Glycyrrhetinic acid (GA, purity 98%) was obtained from Fujie Chemical Co., Ltd. (Xi'an, China). Poly (ethylene glycol) (MW = 2000, PEG_2000_), 1-(3-dimethylaminopropyl)-3-ethylcarbodiimide hydrochloride (EDCI), and dimethylaminopyridine (DMAP) were purchased from Sigma (St. Louis, MO, USA). Cholesterol (Chol) was obtained from Shanghai Bio Life Science & Technology Co., Ltd. (Shanghai, China). All other chemicals were of analytical grade.

### 2.2. Synthesis of GA-PEG-GA Conjugates

**Scheme 1:**
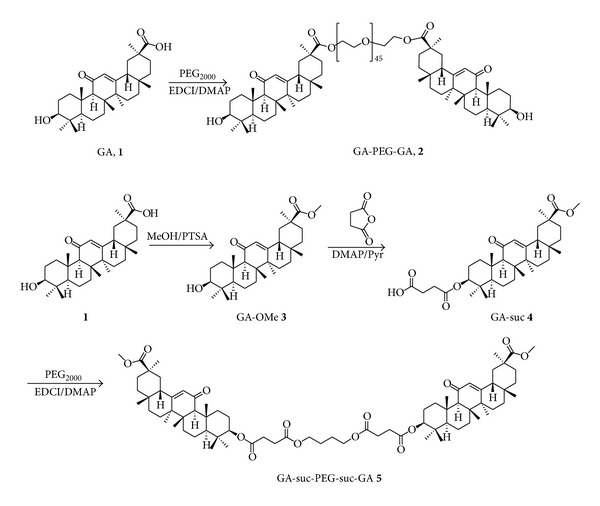
Synthesis of GA-PEG-GA and GA-suc-PEG-suc-GA conjugates.

The synthesis process of GA-PEG conjugates was displayed in Scheme 1. Synthesis of glycyrrhetinic acid methyl ester (GA-OMe, **3**) and glycyrrhetinic acid methyl ester 3-O-hemisuccinate ester (GA-suc, **4**) was carried out according to the literature [19, 24]. The synthesis process of GA-PEG-GA and GA-suc-PEG-suc-GA was briefly described as follows. 

GA-PEG-GA: 18*β*-glycyrrhetinic acid (4.0 g, 8.5 mmol), PEG_2000_ (6.8 g, 3.4 mmol), DMAP (1.0 g, 8.19 mmol), and EDCI (7.8 g, 40.9 mmol) were dissolved in 60 mL of dichloromethane. The solution was stirred viciously and refluxed at 39°C for 5 h. After the reaction was completed, the solvent was removed by evaporation. The residue was dissolved in 20 mL of dichloromethane and washed twice with 1 mol/L HCl to remove DMAP. The combined organic layer was dried with anhydrous Na_2_SO_4_ and concentrated by rotary evaporation. The crude product was purified on a silica gel chromatography column, eluting with gradient ratio of dichloromethane/methanol from 100 : 1 to 10 : 1. The final product (8.7 g, yellow tax) was obtained. The ^1^H NMR spectrum of GA-PEG-GA was characterized using Bruker Avance 400 spectrometer (400 MHz), and CDCl_3_ was used as the solvent. The samples were scanned from 400 to 4000 cm^−1^. ^1^H NMR (400 MHz, CDCl_3_) *δ*5.77 (s, 2H, 12-H∗2), 3.64–3.76 (m, 180H, PEG_2000_, CH_2_∗90), 2.80 (dt, *J *= 13.6, 4.5 Hz, 1H, 1*β*-H), 2.37 (s, 2H, 9*α*-H∗2), 1.37 (s, 6H, CH_3_∗2), 1.15 (s, 6H, CH_3_∗2), 1.14 (s, 6H, CH_3_∗2), 1.13 (s, 6H, CH_3_∗2), 1.01 (s, 6H, CH_3_∗2), and 0.81 (s, 12H, CH_3_∗4). The infrared spectra were also measured using an IR spectrometer (Nicolet 5DX FTIR). IR (film) *ν* (cm^−1^): 2886, 2696, 2239, 1727, 1658, 1146, and 1113.

 GA-suc-PEG-suc-GA: the GA-suc-PEG-suc-GA conjugates (**5**) were prepared from dissolving 2.00 g (3.44 mmol) of **4** in 50 mL of dichloromethane. Then 0.42 g (3.44 mmol) of DMAP and 0.72 g (3.78 mmol) of EDCI and 3.54 g (1.77 mmol) PEG_2000_ were added. The mixture was stirred for 12 h at room temperature, then DMAP and EDCI were washed off by 1 mol/L HCl. The organic phases were collected and dried by Na_2_SO_4_ and the solvent was evaporated *in vacuo*. The products (**5**) were purified on a silica-gel column, eluting with a mixture of DCM-methanol (60 : 1). White solid (2.4 g, 27%) was obtained as ^1^H NMR (400 MHz, CDCl_3_) *δ*5.77 (s, 2H, 12-H∗2), 3.70 (s, 6H, –OCH_3_∗2), 3.64–3.76 (m, 180H, PEG_2000_, CH_2_∗90), 2.80 (dt, *J *= 13.6, 4.5 Hz, 1H, 1*β*-H), 2.51–2.73 (brs,8H, suc, COCH_2_∗4), 2.37 (s, 2H, 9*α*-H∗2), 1.37 (s, 6H, CH_3_∗2), 1.15 (s, 6H, CH_3_∗2), 1.14 (s, 6H, CH_3_∗2), 1.13 (s, 6H, CH_3_∗2), 1.01 (s, 6H, CH_3_∗2), and 0.81 (s, 12H, CH_3_∗4); IR (film) *ν* (cm^−1^): 2741, 2696, 1969, 1732, 1659, and 1147.

### 2.3. Determination of Critical Micelle Concentration (CMC)

The CMC of mPEG-Chol, GA-PEG-GA, and GA-suc-PEG-suc-GA was determined according to the literature [19]. Pyrene probe was used as a probe during CMC determination. The work was performed according to the characteristic of pyrene emission spectrum, a red shift of the band from 373 nm to 384 nm after being encapsulated into a micellar hydrophobic core. Appropriate amount of pyrene dissolved in acetone was added into clean flask and dried by nitrogen instrument. The dried mixtures were hydrated with 10 mL mPEG-Chol or GA-PEG-GA or GA-suc-PEG-suc-GA water solution at gradient concentrations. All the samples were detected on fluorescence spectrophotometer with excitation wavelength at 335 nm and emission wavelengths at 373 nm (I1) and 384 nm (I3). The CMC value was taken from the intersection of the tangent to the curve at the inflection with the horizontal tangent through the points at low concentrations.

### 2.4. Micelle Formation and Drug Loading

GA-PEG-GA micelles (GA-M) were prepared using thin film hydration method. To prepare paclitaxel loaded GA-PEG-GA micelles (GA-M-PTX), 1.0 mg of paclitaxel and 5 mg GA-PEG-GA were dissolved in 5 mL mixed solvent of acetone and chloroform (v/v = 1 : 4) at room temperature. The solvents were evaporated under vacuum at 37°C for 30 min to form a dry drug-containing lipid film. The formed dried lipid film was hydrated with 20 mL Mili-Q water at 40°C and then sonicated in water bath for 30 min. The micelle was centrifuged at 1500 rpm for 10 min and extruded through 220 nm filter to remove unloaded drugs. The final amount of capsulated paclitaxel was measured by high-performance liquid chromatography (HPLC) analysis. 

The coumarin loaded micelles (GA-M-Cou) were prepared by similar method of GA-M-PTX, and the ratio of coumarin to GA-PEG-GA was 1 to 200 (w/w).

mPEG-Chol micelle (Chol-M-Cou) was prepared by dropping the solution of mPEG-Chol and coumarin in DCM (2 mL) into 20 mL Mili-Q water and stirred overnight. The micelle solution was evaporated for 30 min to remove organic solvents. The amount of mPEG-Chol and coumarin used was the same to GA-M-Cou.

### 2.5. Size and Zeta-Potential Determination

Particle size and zeta potential of the micelle were determined by dynamic light scattering (DLS) with a Zetasizer Nano ZS-90 instrument (Malvern Instruments, Malvern, UK). Refractive index was 1.330 and temperature was kept at 25°C during measuring process. The micelle suspension was kept at 25°C during measuring process. All tests were run 3 times and took mean values.

### 2.6. Transmission Electron Microscopy (TEM)

The morphology of PTX-loaded micelles was observed by TEM (H-600, Hitachi, Japan). Before analysis, the samples were diluted 1 : 5 and negatively stained with 2% (w/v) phosphotungstic acid for 30 s and then placed on copper grids precoated with a thin film of polyvinyl formaldehyde for observation. 

### 2.7. *In Vitro* Drug Release

The release profile of paclitaxel from micelles was investigated using a dialysis method. The test was performed on a thermostatic shaker. Briefly, 4 mL GA-M-PTX solution was placed in a dialysis bag (molecular weight cutoff = 1.0 kDa), which was suspended in 150 mL PBS (pH 7.4 0.1 M) with 0.2% Tween-80 at 37°C with shaking at a speed of 100 r/min. 1 mL aliquots were withdrawn and replaced with the equal volume of fresh medium at appropriate time intervals. HPLC was performed to determine the concentration of PTX in recovered release medium. 

### 2.8. *In Vitro* Cytotoxicity Assay

Three kinds of tumor cell (Hela and HepG2) were chosen for the treatment experiment and cultured in DMEM medium containing 10% FBS. Cellular cytotoxicity of blank GA-PEG-GA micelles, paclitaxel loaded GA-PEG-GA micelles (GA-M-PTX), and paclitaxel loaded mPEG-Chol micelles (Chol-M-PTX) were evaluated by MTT (3-[4,5-dimethylthiazol-2-yl]-2,5-diphenyl tetrazolium bromide) assay. Cells were seeded in 96-well plates at a density of 3 × 10^3^ cells per well in 100 uL DMEM. After 24 h of incubation at 37°C with 5% CO_2_, cells were treated with different concentrations of blank micelle, Chol-M-PTX, and GA-M-PTX solution (each well with a ratio of medium component to nonmedium component equivalent to 9 : 1), respectively. 48 hours later, 20 uL MTT (5 mg/mL, dissolved in physiologic saline) was added to each well and incubated for another 4 h. Then the incubated medium was removed, added 150 uL DMSO to each well, and gently shook for 10 min at room temperature. Absorbance was measured at 570 nm using a Spectramax M5 Microtiter Plate Luminometer (Molecular Devices, USA).

### 2.9. Cellular Uptake Experiment

To evaluate the ability of GA-PEG-GA micelles binding to heptoma cells (hepG2), a micelle made of mPEG-Chol was used as a negative control. The cellular uptake tests of GA-PEG-GA micelle and mPEG-Chol micelles were performed by microscope and flow cytometric assay. HepG2 cells were seeded in 6-well plates at a density of 2 × 10^5^ cells per well and cultured for 24 h in 1.8 mL DMED supplemented with 10% fetal bovine serum (FBS), at 37°C in 5% CO_2_. 200 uL of GA-M-Cou and Chol-M-Cou solution loading the equal quantity (0.1 ug per well) of coumarin was added to predesigned wells. After incubation for 3 h and 6 h, respectively, medium was removed, and cells were washed with physiological saline 3 times. The fluorescence of coumarin that entered into the tumor cells was observed through a fluorescence and light microscope (Olympus IX71, Olympus, Japan). Then cells were lysed with Trypsin and collected. Fluorescence intensity of coumarin was measured by flow cytometry.

### 2.10. Tissue Distribution of Coumarin Loaded GA-PEG-GA Micelles

The rats received 1 mg/kg of coumarin loaded GA-PEG-GA micelles and equivalent amount of the mixture of free coumarin and blank GA-PEG-GA micelles in normal saline intravenously, respectively. The rats were sacrificed at predetermined time points and intestines were immediately dissected to expose the main organs before imaging. After intravenous injection, fluorescence imaging was recorded after 4 hours and the real-time images were performed in macroimaging system LT-9 equipped with illumatool dual light system LT-99D2 (Lightools Research, Encinitas, CA, USA).

## 3. Results and Discussion

### 3.1. Synthesis and Characterization of GA-PEG-GA Conjugates

The GA-PEG-GA and GA-suc-Peg-suc-GA conjugates were successfully prepared by using the synthetic route as shown in Scheme 1. The synthesis process of methoxyl-poly (ethylene glycol)-cholesterol conjugate (mPEG-Chol) was reported in our previous studies and other literature studies [26–29]. The method of choice for coupling bioactive components to the PEG backbone is mainly by esterification. This type of esterification can be divided into two main approaches: (a) activation of the hydroxy end group through transformation into a good leaving group and subsequent attack by the carboxylate component, such as the formation of PEG-isourea and PEG-tosylate and (b) activation of the carboxy component and subsequent attack by PEG hydroxy end groups, such as the direct coupling by carbodiimide. Particularly, it was demonstrated that DMAP catalyzed attachment of carbodiimide-activated glycyrrhetinic acid proceeds good yield with PEG under very mild conditions. Therefore, we employed this EDCI/DMAP method in the synthesis of both PEG-amino acid derivatives and the final glycyrrhetinic acid-PEG-glycyrrhetinic acid conjugates.

**Figure 1:**
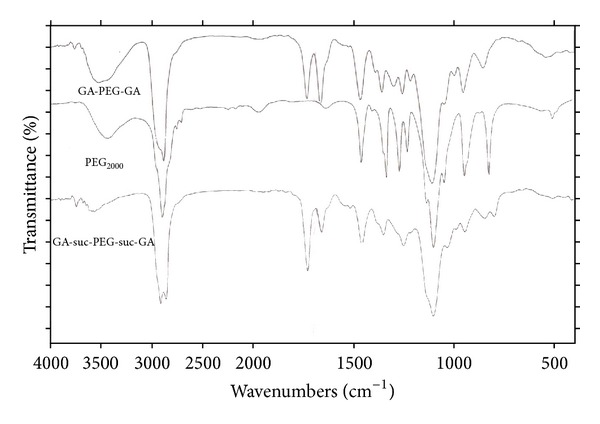
FT-IR spectra of PEG_2000_, GA-PEG-GA, and GA-suc-PEG-suc-GA.

The FT-IR spectra of PEG, GA-PEG-GA, and GA-suc-PEG-suc-GA were shown in Figure 1. The broad band at around 3480 cm^−1^ attributed to the inter- and intramolecular hydrogen bonding of –OH stretching vibration of 3-OH of glycyrrhetinic acid in GA-PEG-GA, because the corresponding hydroxyl groups in PEG_2000_ were primary hydroxyl groups, and the band had moved to around 3390 cm^−1^. The strong peaks around 2880 cm^−1^ were assigned to the –CH_2_– which was brought by PEG. The sharp peak at 1112 cm^−1^ was assigned to the C–O group which was brought by the PEG. Another small peak at 1730 cm^−1^ belongs to the carbonyl group of ester linkage between glycyrrhetinic acid and PEG. Other prominent peaks at 1340 cm^−1^ and 1460 cm^−1^ were assigned to the asymmetrical and symmetrical bending vibrations of methyl, methylene groups which were the introduction of long PEG chain.

**Figure 2:**
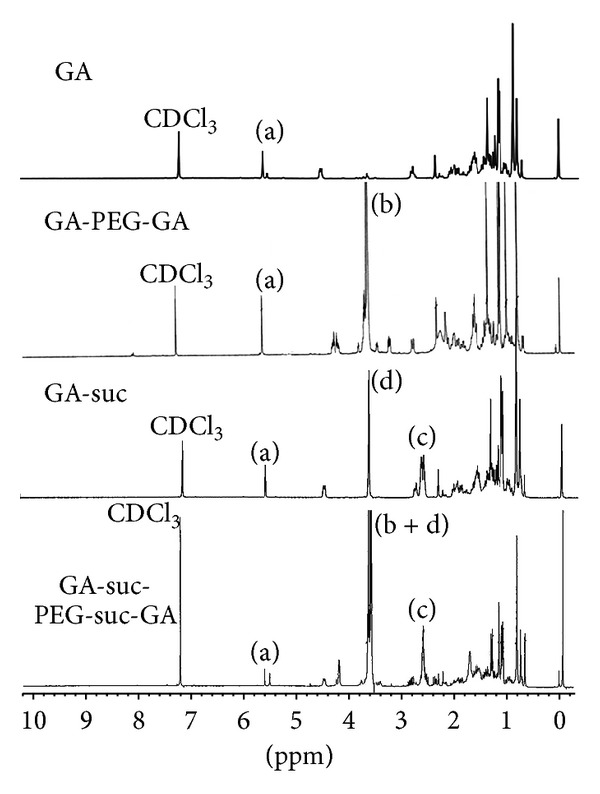
^1^H NMR spectra of GA and GA-PEG-GA and GA-suc-PEG-suc-GA.

Figure 2 showed the ^1^H NMR spectra of GA, GA-PEG-GA and GA-suc-PEG-suc-GA in CDCl_3_. The single peak at *δ*5.69 (a) was attributed to the protons of olefinic bond (–(C=O)–CH=C–) in GA. The peaks at *δ* 3.52–3.76 ((b) and (d)) were attributed to the protons from the glycol unit (–CH_2_–CH_2_–O–) in PEG chain and the protons from methyl ester. The peaks at *δ* 2.58–2.66 (c) came from the protons of succinate linkage (–(C=O)–CH_2_–CH_2_–(C=O)–). Thus the GA-PEG-GA conjugates have been successfully synthesized.

### 3.2. Determination of Critical Micelle Concentration (CMC)

**Figure 3:**
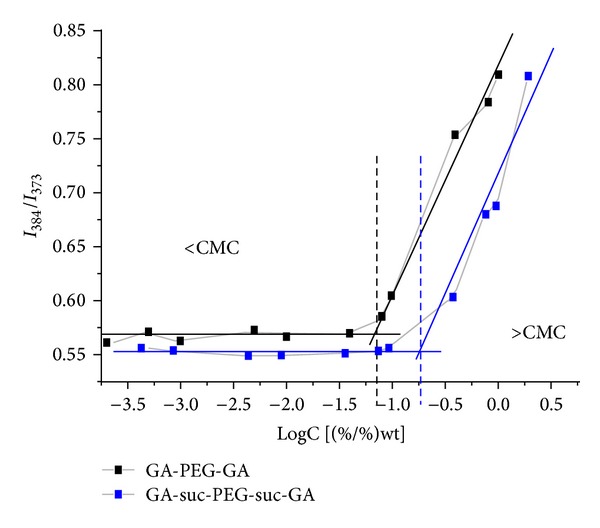
The CMC (critical micelle concentration) of GA-PEG-GA and GA-suc-PEG-suc-GA.

The CMC is an important characteristic for amphiphilic copolymer, demonstrating the self-aggregation of copolymer. To determine the CMC of GA-PEG-GA and GA-suc-PEG-suc-GA, the transition of emission wavelength of pyrene between being free in water and entrapped into micelle was detected.  Figure 3 was made with *I*_3_/*I*_1_ as the *Y*-axis and logC as the *X*-axis, and an inflection point was shown by value of logC. The CMC value of GA-PEG-GA and GA-suc-PEG-suc-GA was shown to be as low as 6.0 × 10^−5^ M and 3.1 × 10^−4^ M, respectively, which was similar to the CMC values of PEG_2000_-PE and mPEG_2300_-DSPE determined by the method of pyrene fluorescence probe and mPEG_2200_-cholesterol determined by the method of surface tension [23–25]. These results indicated that the GA-PEG-GA micelles had lower CMC and higher stability and thereby might retain the integrity even upon strong dilution during systemic circulation. Therefore, the GA-PEG-GA micelles were chosen to perform the subsequent experiments.

### 3.3. Preparation and Characterization of Blank and Drug Loaded GA-PEG-GA Micelles

**Figure 4:**
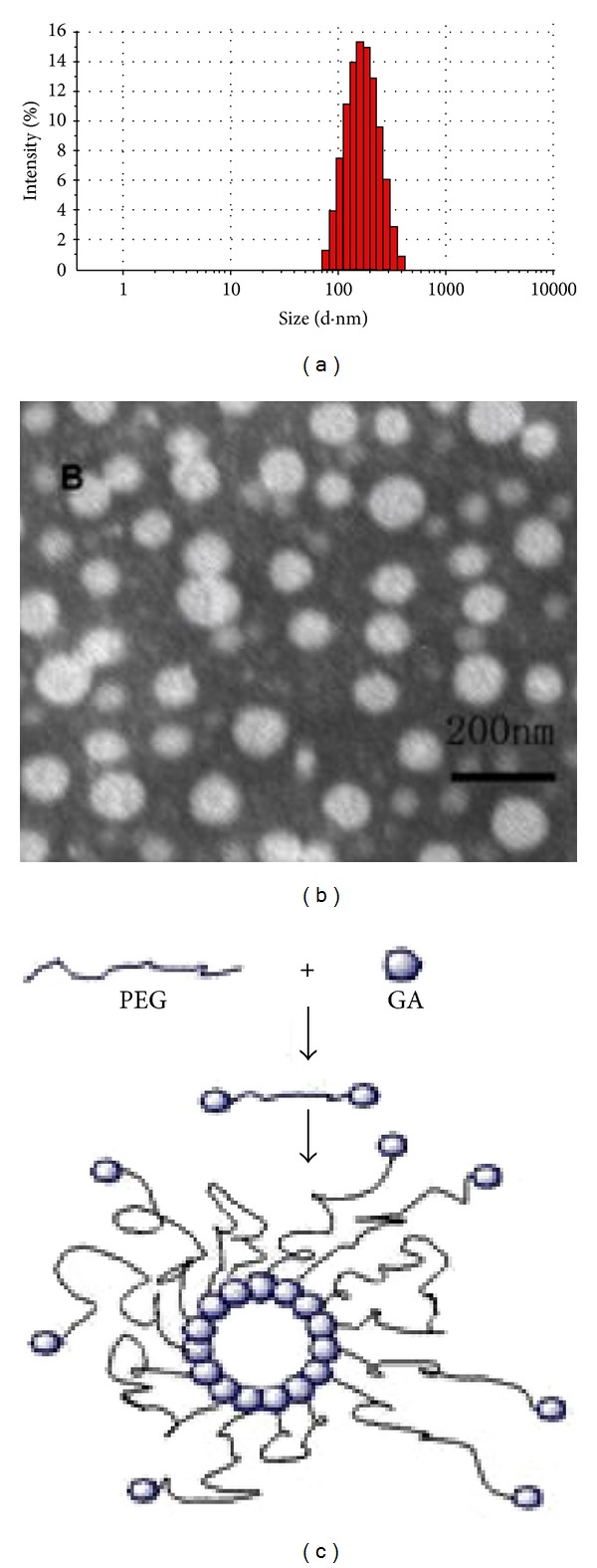
Preparation and characterization of the paclitaxel loaded GA-PEG-GA micelles. (a) Size distribution of the paclitaxel loaded GA-PEG_2000_-GA micelles (GA-M-PTX); (b) TEM image of GA-M-PTX (size of the scale bar: 200 nm); (c) diagram of the preparation of GA-PEG-GA micelles.

The PTX loaded GA-PEG-GA micelles were found to have a mean diameter of 159.21 ± 2.22 (mean ± SD; *n* = 3), with a distribution from 78.8 to 396.1 nm (PDI = 0.123) (Figure 4(a)), which, compared with blank micelle, showed a slight increase in micelle size. The surface charges of GA-M-PTX were −18.53 ± 0.80 mV (mean ± SD; *n* = 3) in zeta potential measurement, which demonstrated the high stability of GA-M-PTX in water. Polydispersity in the micelle diameter can be attributed to a molecular weight distribution. The micelle size represented in the TEM images appeared to be smaller than that detected by DLS (Figure 4(b)). The scheme for the preparation of the GA-PEG-GA micelles was displayed in Figure 4(c). Hydrodynamic radii of particles determined using DLS are typically found to be significantly larger than those determined by TEM. This is seen because the particle radius is based on the intensity of scattered light so the size is skewed toward larger particles. One additional possibility for this difference is the inability to view the corona of the micelle. One would see the corona if the micelles were very densely packed. 

Encapsulating the insoluble drug is an important function of micelles. To evaluate the characteristic of micelle loading PTX, 1 mL methanol was added into 1 mL GA-M-PTX solution and sonicated for 30 min. HPLC was performed, and the quantity of PTX was determined based on a linear standard curve obtained at a concentration range of 0.002–0.01 mg/mL.

The gradient ratio of PTX/GA-PEG-GA used in formulation was determined. When the ratio of PTX to GA-PEG-GA was 1 : 5, the loading efficiency and loading content reached an optimal value, which were equivalent to 59.8% ± 1.4% (mean ± SD) and 13.0% ± 0.3% (mean ± SD).

### 3.4. *In Vitro* Release Profile of Paclitaxel from GA-PEG-GA Micelle

**Figure 5:**
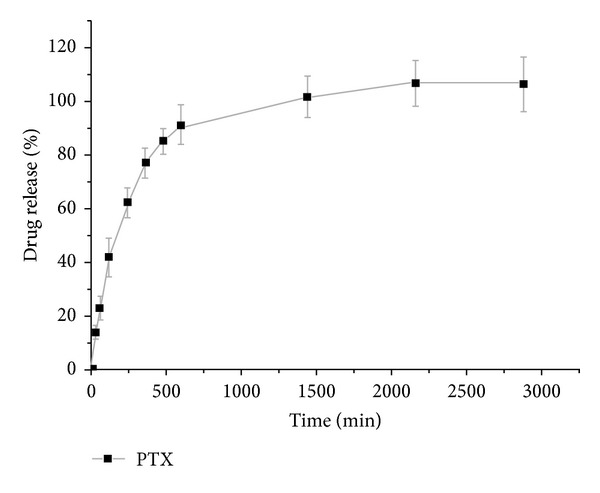
*In vitro* release profiles of PTX from GA-PEG-GA micelles in PBS at pH 7.4.

The *in vitro* release profile of paclitaxel from GA-PEG-GA micelles (GA-M-PTX) was studied at 37°C and pH 7.0. Data suggests that paclitaxel can be well encapsulated in GA-PEG-GA micelles and released in an extended period. As shown in Figure 5, after 10 h, 91.49% of total paclitaxel was released from GA-M-PTX, followed by complete release after 36 h.

### 3.5. *In Vitro* Cytotoxicity Assay

**Figure 6:**
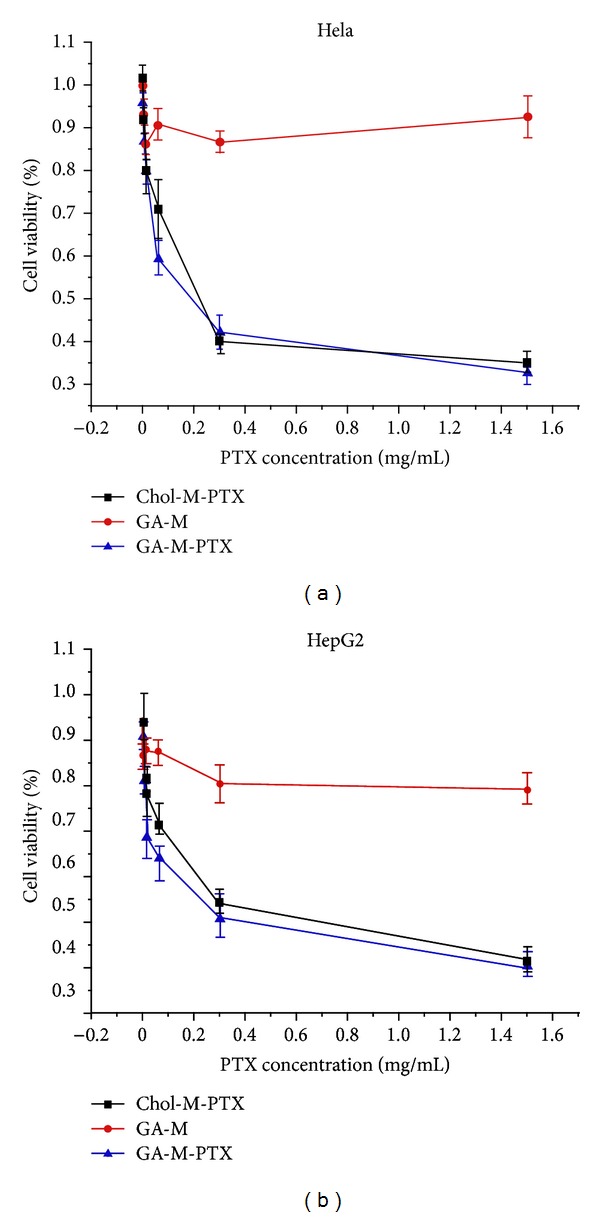
Cytotoxicity of Chol-M-PTX (▪), blank micelles (•), and GA-M-PTX (▴) on Hela (a) and HepG2 (b) cell lines. The percentage of viable cells was quantified using the MTT method. Mean values and 95% confidence intervals derived from three independent experiments are shown. *P* < 0.05.

To evaluate the cytotoxicity of PTX loaded GA-PEG-GA micelles, HpeG2 and Hela cell lines were chosen for MTT analysis. As shown in Figure 6, PTX loaded GA-PEG-GA micelles were comparable to blank GA-PEG-GA micelles and PTX loaded mPEG-Chol micelles in antitumor activity in both cancer cell lines (HepG2 and Hela), and there was no significant difference of cytotoxicity observed between GA-M-PTX and Chol-M-PTX on Hela cell line, the IC_50_ values were 0.22 ug/mL for Chol-M-PTX and 0.20 ug/mL for GA-M-PTX, respectively (*P* < 0.01). As shown in Figure 6(b), GA-M-PTX showed slightly higher cytotoxicity than Chol-M-PTX. The mean concentrations of paclitaxel that caused 50% cell inhibition (IC_50_) of GA-M-PTX were decreased to 0.32 ug/mL compared with 0.54 ug/mL of Chol-M-PTX, respectively. The drug-free GA-M did not show obviously cytotoxicity to the two cells.

### 3.6. Cellular Uptake Experiment

To evaluate the effect of glycyrrhetinic acid block in micelle binding to hepatoma cells, mPEG-Chol micelle (Chol-M) was made as a negative control. HepG2 cells were treated with both micelles (GA-M and Chol-M) encapsulating with coumarin. After incubation for specific time, cells were washed and identified using a fluorescence microscopy. Then cells were collected, and the intensity of coumarin was measured by flow cytometric assay. 

**Figure 7:**
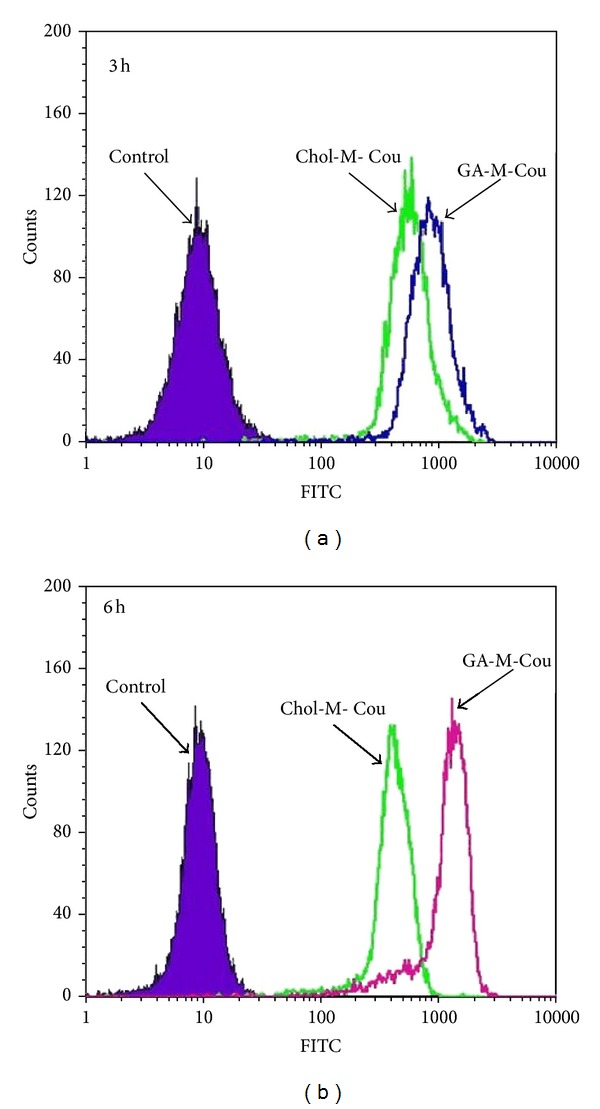
Flow cytometry profiles of hepG2 cell line after 3h (a) and 6h (b) incubation with control, Chol-M-Cou, and GA-M-Cou.

**Figure 8:**
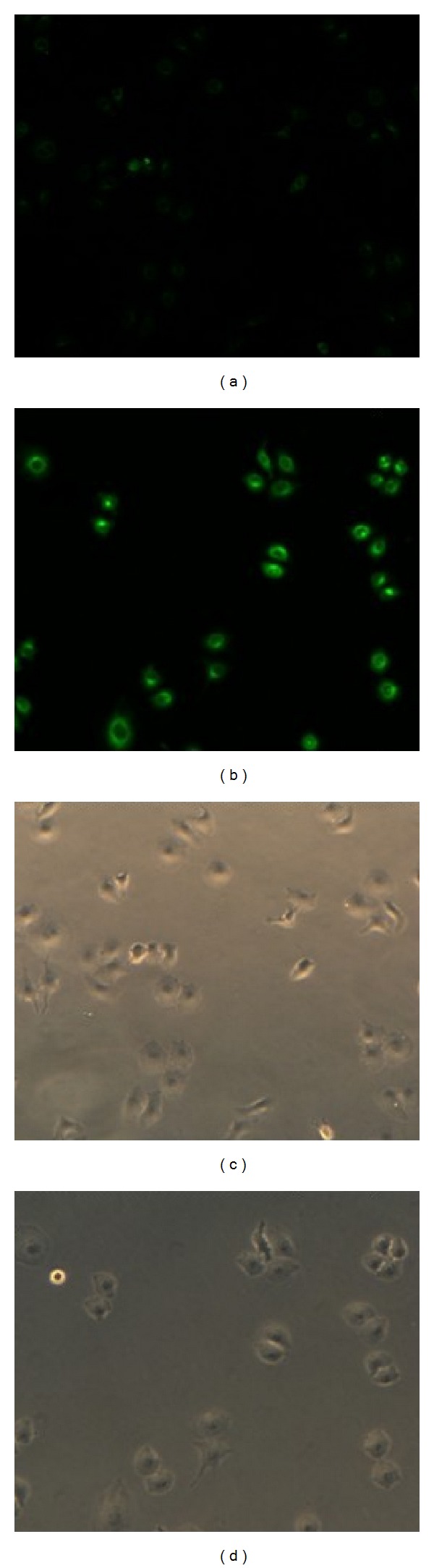
The fluorescence microscopy images and white light microscopy images of HepG2 cells after 6 h incubation with different coumarin loaded micelles: the Chol-M-Cou ((a) and (b)); the GA-M-Cou ((c) and (d)).

The flow cytometry data (Figure 7) shows the distribution of the intensity of coumarin encapsulated into HepG2 cells after with incubated with GA-M-Cou, Chol-M-Cou, or empty GA-PEG-GA micelles (control). The mean fluorescence intensity of coumarin uptaken by cells after 3 h and 6 h incubation clearly showed that the difference of fluorescence intensity between GA-M-Cou and Chol-M-Cou for 6 h incubation become much greater compared with the ones for 3 h incubation. The percentage of positive cells containing coumarin after 6 h incubated with GA-M-cou was significantly increased by 5.1-fold in comparison to that incubated with Chol-M-Cou with the same time (Figure 7(b)). After HepG2 cells were treated with Chol-M-Cou and GA-M-Cou, respectively, according to Figure 8, fluorescence microscopy images showed that GA-M-Cou micelles displayed obviously higher coumarin fluorescence in HepG2 cells than the Chol-M-Cou micelles. 

These results indicated that GA-M-Cou has a better ability of binding to HepG2 than Chol-M-Cou and the treatment time-dependent GA-M-Cou uptakes behavior of HepG2. These results suggest that the glycyrrhetinic acid fragment of the corresponding block copolymer indeed enhances micelles binding to liver cells. 

### 3.7. Tissue Distribution of GA-PEG-GA Micelle

**Figure 9:**
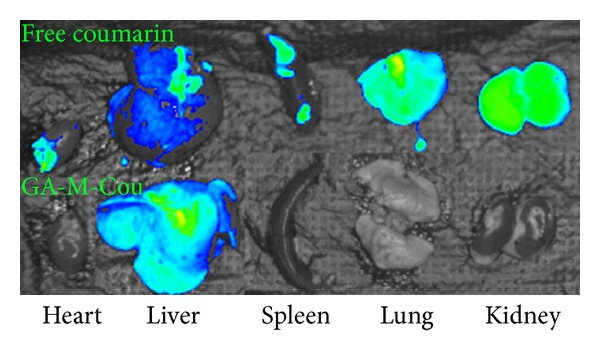
The tissue distribution of free coumarin and coumarin loaded GA-PEG-GA micelle.

As shown in Figure 9, there were significant differences in tissue distribution between free coumarin and GA-M-Cou in rats. The liver was the only tissue with detectable fluorescence after injection of coumarin loaded GA-PEG-GA micelles intravenous. Administration of the mixture of free coumarin and blank GA-PEG-GA micelles resulted in a nonspecific tissue distribution of fluorescence (Figure 9, upper row), confirming that the hepatic selectivity of GA-PEG-GA micelles was indeed attributed to the GA fragment. 

## 4. Conclusions 

In conclusion, the convenient, economic, and effective methods to prepare hepatic targeting polymeric micelles, the surfaces of which are anchored with GA, have been successfully developed for hepatic targeted drug delivery both *in vitro *and* in vivo*. Poorly soluble antitumor drug paclitaxel as model is loaded into the micelles. The micelles are spheroids with regular shape and have a size distribution of about 150 nm and slightly negative surface charge. Flow cytometry results and fluorescence spectroscopy images suggest that the presence of glycyrrhetinic acid on the surfaces of the micelles promotes their uptakes by hepatoma cell lines. Thus the paclitaxel loaded GA-PEG-GA micelles have enhanced cytotoxicity of paclitaxel for hepatocellular carcinoma cells, which was confirmed by MTT experiment. Although further investigation on the *in vivo* antitumor effect of GA-PEG-GA micelles is required, the findings of our study represent an important step in advancing the use of GA-PEG-GA micelles as a potent strategy to treat hepatic carcinoma.
